# Toxicity of particles emitted by fireworks

**DOI:** 10.1186/s12989-020-00360-4

**Published:** 2020-07-02

**Authors:** Christina Hickey, Christopher Gordon, Karen Galdanes, Martin Blaustein, Lori Horton, Steven Chillrud, James Ross, Lital Yinon, Lung Chi Chen, Terry Gordon

**Affiliations:** 1 Department of Environmental Medicine, NYU School of Medicine, 341 East 25th St,, New York, NY 10010 USA; 2 Lamont-Doherty Earth Observatory of Columbia University, Palisades, NY 10983 USA

**Keywords:** Fireworks, Particles, Inhaled metals, In vitro, In vivo, Air pollution

## Abstract

**Background:**

Particle matter (PM) has been associated with increased morbidity and mortality rates across the world. This study was designed to test the hypotheses that pyrotechnic firework displays introduce significant amounts of toxic metals into the atmosphere and are hazardous to human health. Size-selective emissions from 10 different fireworks displays were collected during particle generation in a dynamic, stainless steel chamber and tested for toxicity in cells. A subset of 2 particle types were tested in vivo in mice. At doses that did not produce cytotoxicity in an LDH assay, in vitro reactive oxygen species (ROS) formation was measured in bronchial epithelial airway (BEAS-2B) and human pulmonary microvascular endothelial (HPMEC-ST1.6R) cell lines treated with size-fractionated particles from the emissions of fireworks.

**Results:**

Significant increases in ROS, in both cell types, were dependent upon the type of firework but not particle size. The in vitro ROS activity was correlated with lung inflammation produced in groups of mice treated by oropharyngeal aspiration with 0, 50, or 100 μg fireworks PM_10_/mouse. Trace metal analyses of the PM_10_ samples showed significant differences in metal content among fireworks type. Interestingly, the PM_10_ sample for the fireworks type producing the greatest in vitro ROS response in BEAS-2B cells contained ~ 40,000 and ~ 12,000 ppm of lead and copper, respectively. This sample also produced the greatest inflammatory response (i.e., increased neutrophils in bronchoalveolar lavage fluid) in mice.

**Conclusions:**

These findings demonstrate that pyrotechnic display particles can produce adverse effects in mammalian cells and lungs, thus suggesting that further research is needed to expand our understanding of the contribution of metal content to the adverse health effects of fireworks particles. This information will lead to the manufacture of safer fireworks.

## Background

Throughout the year, numerous types of celebratory fireworks or pyrotechnic displays are set off across the world. Often, the only limit on the size and number of the displays is cost. In the past, while U.S. pyrotechnics traditionally were reserved for special occasions such as July 4th and Chinese New Year, pyrotechnic displays are now prevalent at rock concerts, opening ceremonies of the Olympics, amusement parks, and sports venues. Amusement parks are the largest consumers of fireworks in the U.S., whereas the single largest fireworks show is the July 4th display sponsored by Macy’s [1]. In addition to these large public fireworks displays, small, and often illegal, fireworks are often ignited locally within residential neighborhoods. According to the American Pyrotechnics Association, the amount of consumer fireworks (258.4 million pounds) intended for use by the general public (i.e., 1.4G explosives) and purchased in the U.S. is more than 10-fold greater than that used for large celebratory fireworks (19.1 million pounds) displayed by pyrotechnic professionals (i.e., 1.3G explosives) [2] and, thus, are a significant concern for adverse health effects.

To date, the greatest health concern regarding fireworks has been the potential for injury to life and limb due to the explosive force of fireworks. Each year, approximately 10,000 to 25,000 people (predominantly male teenagers) in the U.S. suffer physical and burn injuries, due to fireworks, which include the loss of fingers, limbs, eyesight, and sometimes, life [3–5]. The environmental effects are also a concern as evidenced by numerous publications that describe the release and contamination of air and waterways with perchlorates and other toxicants [6, 7]. Yet, even though there has been a large increase in the amount and size of fireworks events, little to no research has investigated the effect of fireworks-generated particles, and their composition, on human health [6, 8–12]. In fact, epidemiology studies examining the health effects of ambient particulate matter (PM) routinely remove the health and exposure assessment data from celebratory periods, such as July 4th and New Year’s Eve, because of the vast change in source composition of PM at those times. In the present study, the ROS responses of cells to a number of fireworks PM was greater than the typical response to ambient PM ([13]. Interestingly, in our previous in vitro ambient PM study, the highest ROS response of 360 samples, collected in winter and summer at 5 sites across the U.S., was produced by a PM2.5 sample collected on July 4th, 2008 in Anaheim, California (courtesy of Dr. Michael Kleinman, University of California, Irvine [13];). Of note, the collection site in Anaheim was located near a major theme park which sponsors a large firework holiday celebration.

Emissions from pyrotechnic displays are composed of numerous organic compounds as well as metals. The high temperature ignition of different metal compounds, which are purposefully added to fireworks, produces different fireworks colors [14]. While research has addressed the exposure assessment of organic effluents [6, 15] and the contribution of fireworks celebrations to ambient PM composition [16, 17], the toxicity of fireworks has not been explored. This paper describes the in vitro and in vivo toxicity of PM_10_ produced by a number of differently colored pyrotechnic firework displays. The generation of reactive oxygen species (ROS) by airway epithelial and vascular endothelial cell lines was examined in vitro and a subset of fireworks emissions were tested for their ability to produce pulmonary inflammation and injury in an in vivo mouse model. Because the physical-chemical properties of inhaled PM can modify pulmonary toxicity, both trace element composition and particle size were also investigated in this study.

## Materials and methods

### Particle sampling

Ten types of pyrotechnic displays (Table 1) were ignited in a 1 m^3^ stainless steel chamber which was modified so that all incoming air was drawn in through a HEPA filter attached to the top inlet of the chamber. Two collection methods were used to sample airborne particles over a 20 min period or until ‘smoke’ had cleared from the chamber. A stainless steel cyclone was used to impact and remove particles greater than 10 μm in aerodynamic size, and PM_10_ was collected on an 8″ by 10″ polypropylene substrate (GM-3500, Manadnock Non-Wovens, Mount Pocono, PA, USA) using a high volume sampling pump (500 L/min). The cyclone/filter system was calibrated with a Venturi flow meter. Two co-located Sioutas Cascade Impactors (SKC, Inc) were calibrated with a bubble meter (Gilibrator-2, Sensidyne, Clearwater, FL, USA) and operated at 9 L/min to collect size-separated coarse, fine, and pseudo-ultrafine (UF) particles from the chamber (PM_10–2.5_, PM_2.5–0.25_, and PM_0.25_, respectively), for 3 fireworks types, onto 37 mm Teflon filters (Pall Corporation, Port Washington, NY, USA). Although this paper refers to PM_0.25_ as UF, it must be noted that this is due to the impactor specifications and does not match the widely accepted < 0.1 μm definition of UF PM. All collection substrates were acclimated for at least 24 h before being weighed pre- and post-sampling with a Mettler 2500 scale (Toledo-Mettler) in a temperature- and humidity-controlled weighing room. After weighing, filters were stored under sterile conditions at − 80 °C. Particles were then sterilely scraped into pre-weighed sterile polypropylene tubes and diluted with sterile water to 250 μg/ml and 1 mg/ml for the in vitro and in vivo experiments, respectively.

**Table 1:** X-ray fluorescence analysis of fireworks PM_10_. Up to the top 5 trace elements (ppm) are presented for each display type

Product Name	Major Trace Elements^a^	Concentration (ppm) in Collected PM_10_
Purple Colorful Storm	Ti	22,000
	Cu	44,000
	Al	8600
Yellow Colorful Storm	Cu	14,000
	Sr	1200
	Zn	730
Blue Colorful Storm	Cu	53,000
	Ti	11,000
	Al	5200
	Sr	4800
Red Colorful Storm	Al	40,000
	Ba	10,000
	Sr	6200
Color Changing Wheel	Ba	11,000
	Sr	6300
	Ti	3800
Tiger Roaring 1	Ba	430
Tiger Roaring 2	Fe	1200
	Ba	460
Black Cuckoo	Pb	40,000
	Cu	12,000
	Ba	5300
	Al	3100
	Sr	3000
Saturn Missiles 1	Fe	3100
	Pb	1600
	Br	850
	Co	150
Saturn Missiles 2	Fe	4200
	Br	850
	Co	170
Bottle Rocket		Not determined
Firecrackers	Al	95,000
	Fe	6300
	Zn	2000

^a^ Excluding sulfur, potassium, and chloride which were present in all samples at high concentrations (typically greater than 20,000 ppm). Duplicate experiments with 2 fireworks (Tiger Roaring and Saturn Missiles) were conducted to examine variability between commercial samples

### Trace element analyses

Particles were also scraped off the PM_10_ filters into polypropylene tubes and transferred to Lamont-Doherty Earth Observatory for elemental analysis. The powdered samples were transferred into XRF cups normally used for dried sediment samples. Samples were analyzed on an energy dispersive, polarized excitation x-ray fluorescence spectrometer with a 50 W, 50 kV Pd tube as primary source, the radiation of which was modified by four secondary targets to optimize the excitation across the entire elemental range (XEPOS by Spectro Analytical, Kieve, Germany). Helium gas was used to flush the spectrometer allowing light elements to be included with total elemental range from Na to U. Resulting spectra were processed by the manufacturer’s Turboquant™ calibration software which allows for semi-quantitative analysis of a wide range of sample matrices. Without a matrix specific calibration, resulting concentrations are expected to be within 30% of true concentrations although larger differences have been observed between Turboquant concentrations and matrix specific calibrations [18]. However, relative differences of samples with similar matrix are well constrained by the Turboquant software (e.g., can determine that sample A is 2.1 times the concentration of sample B even though the accuracy of the concentration of both samples may both be too low). Detection limits for powdered samples range from 10’s of ppm for light elements such as Na and Mg to around 1–2 ppm or less for a wide range of metals.

### Cell culture

To assess the toxicity on likely lung cell targets of inhaled fireworks particles, 2 cell lines were tested with the PM10 emitted by the fireworks. A human pulmonary microvascular endothelial cell line (HPMEC-ST1.6R) was provided by Drs. James Kirkpatrick and Vera Krump-Konvalinkova (Johannes Gutenberg University, Mainz, Germany). As previously described [19], HPMEC cells were maintained at 37 °C in a humidified atmosphere of 5% carbon dioxide and grown in Endothelial Growth Medium (EGM-2) containing 1% penicillin/streptomycin (Gibco, Grand Island, NY, USA) and supplemented with an EGM-2 BulletKit and 5% fetal bovine serum (Lonza, Switzerland). A bronchial epithelial cell line (BEAS-2B) was obtained from the American Type Culture Collection (ATCC, Rockville, MD, USA) and maintained in DMEM medium (Dulbecco’s Modified Eagle Medium; Gibco) with 10% fetal bovine serum (FBS) (Gemini Bio Products, Calasas, CA, USA) and 1% penicillin/streptomycin (Gibco).

### Lactate dehydrogenase (LDH) and reactive oxygen species (ROS) assays

Cells were seeded onto a COSTAR 96 well plate (Fisher Scientific, Pittsburgh, PA, USA) and the LDH (Takara Bio Inc., Madison, WI) and ROS assays were performed as previously described [19]. After thawing, particles were diluted in cell culture media and initially dispersed in an ultrasonic water bath at a maintained temperature of < 28 °C, for at least 20 min. Immediately before applying to wells, PM samples were vortexed for 10 s to suspend the particles. Sufficient material was available from the impactor sampling of only 3 types of fireworks to examine size-dependent differences in toxicity. For the ROS and LDH assays, cells were washed with PBS (phosphate buffered saline), and the media was changed to DMEM/F12 (Dulbecco’s Modified Eagles Medium/Nutrient Mixture F-12, Gibco) containing no phenol red, 2% FBS, and 1% penicillin/streptomycin prior to treatment with PM. At the tested final PM concentrations of 50 and 100 μg/ml, no cytotoxicity was observed in the LDH assay, so treatment concentrations of 10, 50, and 100 μg/ml were used in the ROS assay. Briefly, for the measurement of ROS, cells were loaded with dichlorofluorescein prior to treatment with particle extracts. After dye removal and washing, the pyrotechnic particles were sonicated for 30 min and added to the cell culture media in each well for a final concentration of 10, 50, and 100 μg/ml, for each particle type (in triplicate). Vanadium and carbon particles with 5% iron (generated by a high voltage spark, a gift of Drs. Oberdorster and Elder, U of Rochester [20];) were used as positive control particles, whereas carbon black (Printex, Orion, Houston, TX) served as a negative control particle. The plates were maintained at 37 °C and 5% CO_2_ and monitored in a fluorescence microplate reader (HTS 7000, Perkin Elmer, Waltham, MA, USA with HTSoft software) every 30 min for 3 h using excitation and emission filters of 480 nm and 535 nm, respectively. ROS production was calculated as the net increase in fluorescence intensity, over time, by subtracting the mean media vehicle blank from the change in fluorescence intensity for each treatment and as the fold increase in ROS activity over the response to control media alone.

### Animal study

Eight to 10 week old male and female FVB/N mice were purchased from The Jackson Laboratory (Bar Harbor, ME, USA) and bred at NYU. Mice were housed in polycarbonate cages with corn-cob bedding in temperature and humidity controlled rooms with a 12 h light/dark cycle. Animals were provided standard chow and water ad libitum. All animal procedures and handling were performed under the National Institutes of Health and Animal Welfare Act guidelines for the ethical treatment of animals using an approved NYU School of Medicine Institutional Animal Care and Use Committee protocol.

A subset of 2 of the 10 extracted PM_10_ samples was selected for an in vivo bioassay of lung inflammation and injury in mice. These samples represented fireworks PM which produced the highest and lowest in vitro ROS response: Black Cuckoo and Roaring Tiger, respectively.

An oropharyngeal aspiration technique [21] was used to disperse PM into the lungs of mice. Briefly, mice were anesthetized with isofluorane (Abbott Laboratories, King of Prussia, PA, USA), placed on a 45° board, and aspirated with 50 μL of sterile water (negative control) or 50 μL of PM suspended in sterile water (vortexed immediately before delivery) for a total dose of 0, 50, or 100 mg/mouse (*n* = 5 per group). Twenty-four hours after aspiration, animals were euthanized with 0.26 mg/g sodium pentobarbital (ip). The lungs were lavaged twice using PBS (Invitrogen) and cell counts, cell differentials, and total protein (BCA Protein Assay Kit, Thermo Fisher Scientific, Pittsburgh, PA, USA) were measured in lavage fluid.

### Statistics

Prism 5.0 for Windows (GraphPad Software, San Diego, CA, USA) was used for the analysis of data, which are reported as means + standard error (SE). Where appropriate, data were analyzed using unpaired t-tests. For in vitro experiments using more than two groups for analysis, a one-way ANOVA, followed by Tukey’s Multiple Comparison Test, was used. For in vivo experiments using more than two groups for analysis, one-way ANOVA, followed by Dunnett’s Multiple Comparison Test, was used. The statistical significance was set for *p* < 0.05.

## Results

### PM characterization

**Fig. 1:**
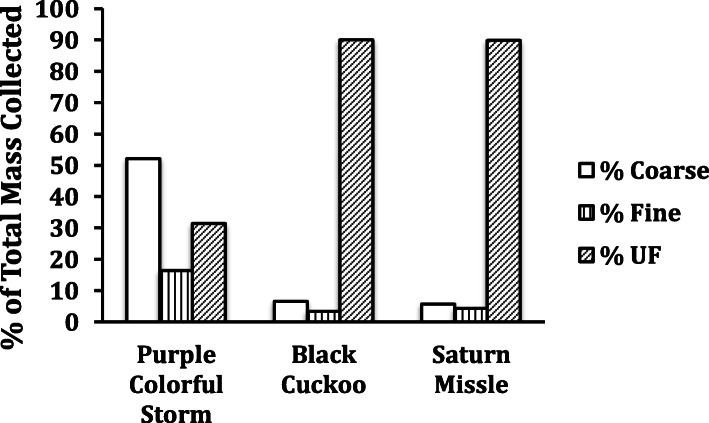
The contribution of each particle size to the total mass, collected by a 3 stage Sioutas cascade impactor, for 3 fireworks types

The total amount of PM_10_ collected varied among fireworks display type, likely due to the differences in display size and purpose (e.g., small missiles flares vs. firecrackers vs. colorful display units) of the purchased products (30–60 g were generated from each fireworks type and PM10 comprised approximately 1/3 of this weight; data not shown). Interestingly, the relative mass of coarse, fine, and UF particles collected varied among fireworks types (Fig. 1). For example, the Back Cuckoo and Saturn Missiles samples produced predominantly UF particles (~ 90%), whereas the Purple Colorful Storm display had a more even distribution of particles with coarse PM accounting for ~ 50% of the collected mass.

The trace element composition varied significantly among the PM10 collected from the fireworks display types. Whereas sulfur, potassium, and chlorine were common major components of the particle emissions of each firework type (data not shown), there were significant differences in the levels of toxic metals (Table 1). In general, high amounts of Fe, Al, Cu, Ba, Ti, and Sr were measured in the PM_10_ of multiple display types. Surprisingly, Pb was present in the PM_10_ of 2 display types with > 40,000 ppm Pb in the Black Cuckoo sample.

### In vitro study

**Fig. 2:**
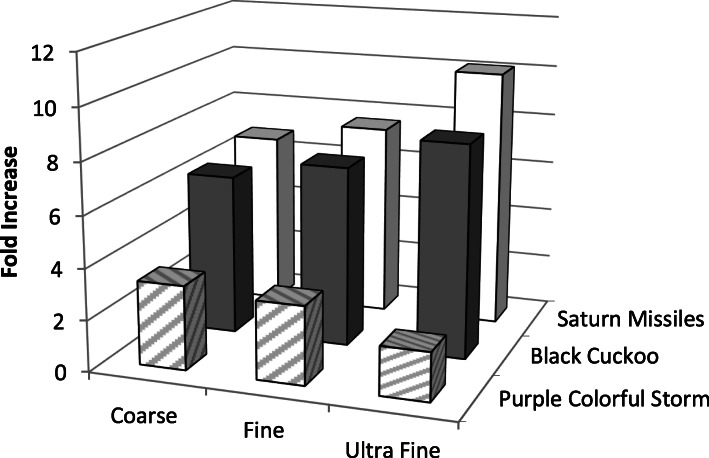
The effect of particle size on ROS activity (measured in triplicate) in BEAS2-B cells (fold increase in fluorescence intensity over media control) treated with 100 μg/ml of 3 selected fireworks particle types for 3.5 h

Cytotoxicity, as measured by the LDH assay, did not occur in either cell line at 100 μg/ml for each display type (data not shown), so this non-cytotoxic concentration was chosen as the high dose in the ROS assay. The ROS results demonstrated that the fireworks type and presumably its composition, played a more important role in ROS activity than did particle size. In a subset of 3 fireworks types, ROS activity in BEAS-2B cells was assessed after treatment with Coarse, Fine, or UF PM (Fig. 2). Particle size did affect ROS activity, but this size-dependence response was influenced by fireworks type. For example, the UF particle size induced less ROS activity than the Coarse and Fine particle sizes in 1 fireworks type (Purple Colorful Storm), whereas the UF particles produced the most ROS activity for the other 2 fireworks types. Regardless of particle size, the Black Cuckoo and Saturn Missiles fireworks produced greater ROS activity than did the Purple Colorful Storm fireworks.

**Fig. 3:**
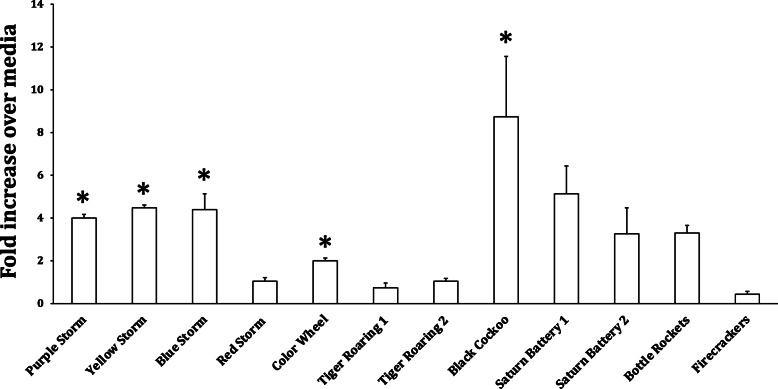
The effect of 12 fireworks types on the fold increase (over media control) in ROS activity in BEAS2-B cells treated with 100 μg/ml (PM10). The columns and error bars represent the mean and SEM, respectively. * *p* < 0.05 compared to media control

**Fig. 4:**
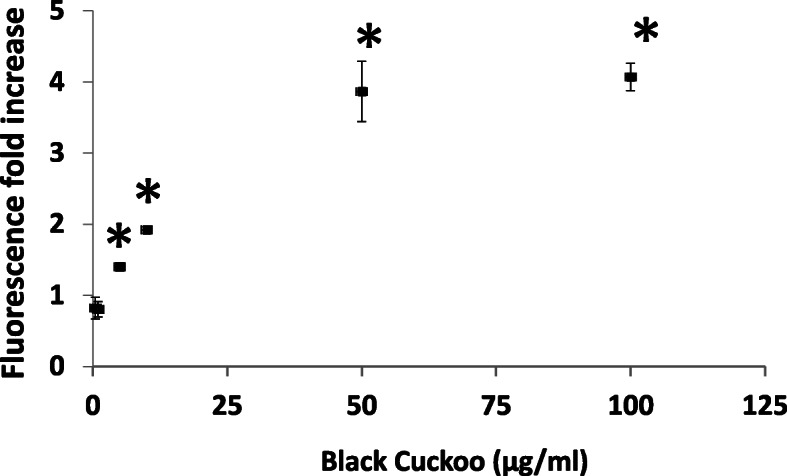
Dose-response change in ROS activity generated in BEAS-2B cells (fold increase in fluorescence intensity over media control) treated with the Black Cuckoo PM10 sample. Squares and error bars represent means ± SE. * *p* < 0.05

The type of fireworks display significantly influenced the ROS activity in BEAS-2B and HPMEC cells challenged with PM_10_ (only BEAS-2B results are shown although the ROS responses in HPMEC cells [Supplemental Fig. 1] were similar). The oxidative stress responsiveness of the cells was confirmed by the significant increases in ROS observed after treatment with the positive control particles comprised of vanadium or carbon with 5% iron. No significant effect on ROS activity was observed for the negative control carbon particles. A subset of the types of fireworks caused a significant increase in ROS response, while other particle types caused only a low or no increase in ROS (Fig. 3). This was best illustrated by the group of 4 Colorful Storm pyrotechnic displays which were identical in construction (i.e., the size and shape of the firework display), but differed in the color and the composition of particles (Table 1) produced by ignition. 100 μg/ml of PM_10_ produced a significant increase in ROS activity in BEAS-2B cells for 3 Colorful Storm samples, whereas the response to the Red Colorful Storm sample was not significantly different from cell culture media alone. It should be noted that this Red Colorful Storm PM10 sample had a much lower copper concentration than the 3 other Colorful Storm samples. Of the 10 product types (2 types were tested in duplicate), the Black Cuckoo PM_10_ produced the largest ROS response in BEAS-2B cells and an extended dose-response curve demonstrated that increased ROS activity occurred at 5 μg/ml (Fig. 4). A regression analysis demonstrated that Cu was the only trace metal statistically correlated with an increase in ROS response in BEAS-2B (*r*^2^ = 0.43; *p* = 0.03) and HPMEC (*r*^2^ = 0.65; *p* = 0.03) cell lines.

### In vivo study

**Fig. 5:**
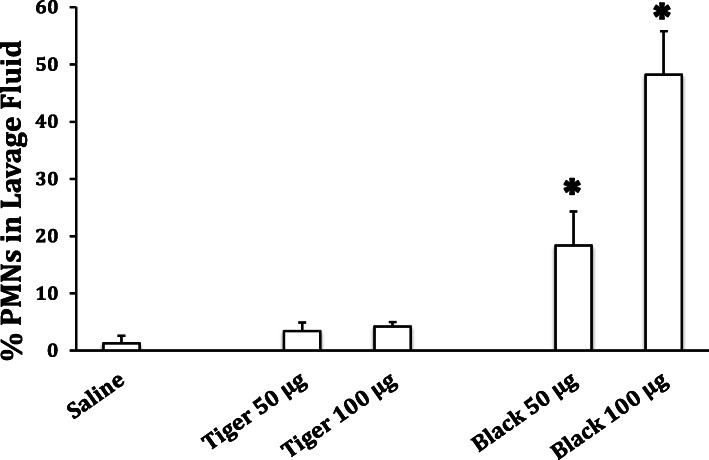
The effect of 2 fireworks types on the influx of inflammatory neutrophil (PMNs) in the lavage fluid of mice (*n* = 5/group) treated with sterile saline or 50 or 100 μg PM_10_ generated from the Black Cuckoo PM10 fireworks. The columns and error bars represent the mean and SEM, respectively. * *p* < 0.05 compared to saline control group

To test whether an in vivo response to fireworks particles was similar to that seen in vitro, using a subset of the fireworks particles tested in vitro, mice were treated with 50 or 100 μg of Tiger Roaring (produced a low in vitro ROS response) or Black Cuckoo (high ROS response) PM_10_ via oropharyngeal aspiration. Twenty-four hours later, lung inflammation (denoted as an increase in neutrophils in lavage fluid) was significantly increased in mice treated with 50 and 100 μg of the Black Cuckoo, but not the Tiger Roaring, particles compared to the response in vehicle-treated (i.e., sterile water) control mice (Fig. 5). These results suggest that the in vitro ROS response has potential as a predictor of what to expect in vivo*.* No significant increases in protein or other cell types were observed (data not shown).

## Discussion

The excitement and pleasure associated with the colors and sounds generated by fireworks are important celebratory components of cultures throughout the world. Yet, the adverse health effects of fireworks have been evaluated almost exclusively for: 1) the injuries and burns produced by the rapid changes in the physical environment attributed to the explosive forces of fireworks; and 2) the release of chemicals that pollute waterways. Injuries to limbs and fingers occur predominantly in males during the teen years and such accidents are recorded and reported at many levels of the health care system in the U.S. [3–5, 22–24]. These acute injuries and burns are significant adverse health effects and largely preventable by the use of proper safety procedures. Little to no research, however, has addressed the potential for the particles and gases released during fireworks celebrations to cause adverse cardiopulmonary effects via the inhalation exposure route [25]. This research study addressed this knowledge gap by examining the in vitro and in vivo toxicity of PM generated by a selection of fireworks displays that are commonly used by individuals at home.

The in vitro experiments clearly demonstrated that particle size had an effect on the ROS activity response in both airway epithelial and vascular endothelial cell lines. Presumably, this was due to differences in particle composition among the ultrafine, fine, and coarse particle sizes. This particle size-dependent effect on cell-based ROS production was accompanied however, by an even greater effect of the type of fireworks being tested. Five of the 10 fireworks products (2 products were assessed in duplicate) had significant increases in ROS activity (Fig. 3) and as shown in Table 1, these fireworks-dependent differences in response were accompanied by significant differences in trace element composition of the PM_10_ collected after ignition of each pyrotechnic display in a stainless steel chamber. On a mass concentration (μg/ml) basis, the ROS effect of these fireworks particles was greater than that of ambient PM collected in the NYC metropolitan area (data not shown). Because of the well established role of metals in oxidative stress [13, 26], the observed differences in metal content among the fireworks types were likely responsible for the fireworks type-dependent differences in ROS activity.

Importantly, fireworks manufacturers adjust the metal content of the each fireworks to produce the desired color based upon the high temperature oxidation of metals. A particular concern in our findings is the disturbing amount of Pb in 2 of the tested fireworks. At 40,000 ppm, it is likely that Pb was not an inadvertent contaminant in the Black Cuckoo PM_10_ sample, but purposely added to the product for achieving the desired effect in the fireworks. Because the 10 fireworks chosen for this study were all pyrotechnic displays that can be purchased by individuals and used at home, the potential for exposure of children to significant amounts of Pb and other toxic metals from such products is unwarranted yet preventable.

There were limitations to the study which impair a full understanding of the contribution of physical and chemical properties to the toxicity of particles emitted by pyrotechnic displays. The study was focused on the pulmonary toxicity of particles generated by fireworks and therefore, thoracic and respirable particle size were collected for the in vitro and in vivo studies. The stability of fireworks particles, by size or otherwise, in the generation chamber were not assessed and nor was the effect of climatic conditions on the particle stability.

**Fig. 6:**
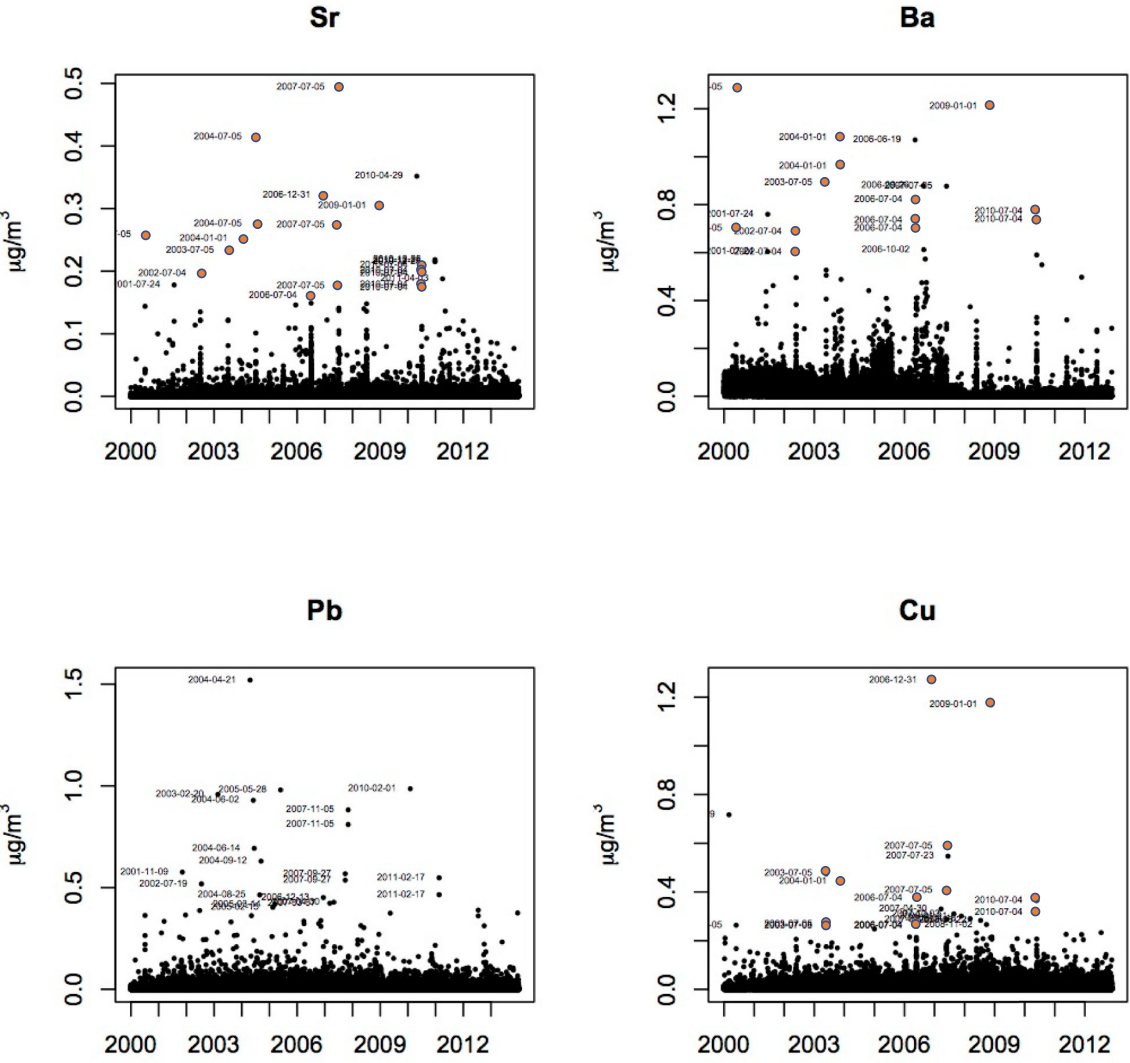
The levels of strontium (SR), barium (Ba), lead (Pb), and copper (Cu) as measured at EPA speciation sites across the U.S. from 1999 to 2014 [27]. The Y-axis is μg/m^3^ as measured in the ambient air. Red circles indicate samples taken on or around holidays (i.e., July 4 or 5; December 31 or January 1)

The results of this study suggest that in addition to organic pollutants [6], significant amounts of toxic metals are released into the ambient environment from both home pyrotechnic displays and larger commercial fireworks. These releases, as demonstrated by exposure assessment air pollution studies [16, 25], are episodic in nature but can potentially be significant emission sources of metals. We have confirmed this time-dependent increase in airborne metal concentrations throughout the U.S. by using a 10 year period of data from EPA’s speciation network [27]. During that period, for example, 19 of the 22 highest peaks (i.e., greater than 0.15 μg/m^3^) for strontium (Sr) in airborne particles in the U.S. occurred on the days surrounding July 4th and New Year’s Day (red circles in Fig. 6). Similar celebration-associated episodic results (Fig. 6) occurred for some (i.e., Ti), but not all (Fe and Pb), of the major trace elements associated with the in vitro and in vivo toxicity observed in this study.

Given the increases in ROS production observed in vitro and the correlated in vivo changes, as well as the episodic elevations in fireworks-associated metals associated with July 4th and New Year’s Eve celebrations (Fig. 6), targeted interventions in fireworks manufacturing can reduce the potential for adverse cardiopulmonary effects in the U.S. and globally. The Walt Disney Company, for example, has addressed contamination concerns in Florida’s Everglades National Park by substituting pneumatic launching systems for explosives charges in their daily fireworks displays. Also, chlorine-free fireworks have recently been developed to replace the blue, green, and red colors produced by chlorine-based stable metal compounds [28]. Similarly, Gluck [28] has suggested that lithium colorant displays can be utilized to replace the red color produced by Sr in fireworks, whereas Han [29] has demonstrated that a new unpacking powder containing ‘micronano’ silicon can reduce the amount of PM released into the atmosphere. In addition, although primarily for reasons of cost, some fireworks exhibitions have been replaced by light shows that encompass laser and LED displays accompanied by music and explosive sounds.

## Conclusions

Our research has demonstrated that fireworks particulate emissions are more toxic in vitro than typical urban particulate matter. This in vitro toxicity was dependent on the composition of the particulate emissions as shown by the large range in toxicity among the fireworks types examined in this study. The in vivo studies in mice validated the in vitro findings for a subset of particles and suggest that the in vitro results are translatable to the mammalian lung. Surprisingly, highly toxic metals, such as Pb, were present at exceedingly high levels in the emissions of some of the tested fireworks. Our temporal survey of the metal species present in the air across the U.S. demonstrated that the metals associated with increased toxicity in our ground-based pyrotechnic displays are elevated in samples taken around the holiday celebrations of July 4 and New Years. These findings bring up the obvious question of whether adverse cardiopulmonary effects are associated with exposure to fireworks-linked metals during these holiday periods. Responsible manufacturing can have a major impact on reducing toxic metals in both commercial and residential pyrotechnics displays and their potential for producing adverse health effects.

## Supplementary information

**Additional file 1: Supplemental Figure 1**. The effect of 12 fireworks types on the fold increase (over media control) in ROS activity in HPMEC cells treated with 100 μg/ml (PM10). The columns and error bars represent the mean and SEM, respectively. * *p* < 0.05 compared to media control.

## Data Availability

The datasets used and/or analysed during the current study are available from the corresponding author on reasonable request.
