# Supplementary figures and images for: Toxicity of particles emitted by fireworks
Source: Part Fibre Toxicol. 2020 Jul 2;17:28. doi: 10.1186/s12989-020-00360-4 (PMC7330945; doi:10.1186/s12989-020-00360-4)

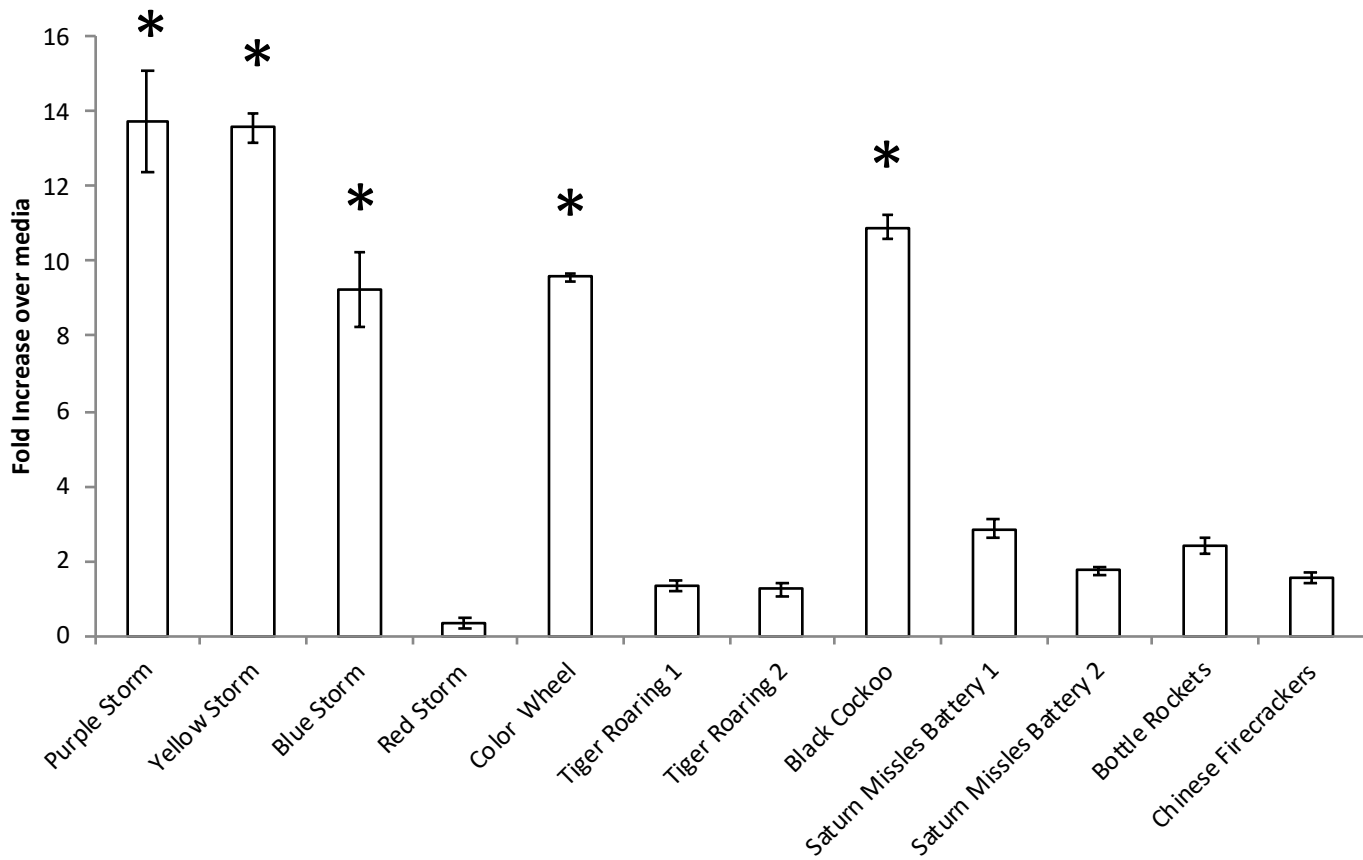

Supplement: Supplementary file 1 — Additional file 1: Supplemental Figure 1. The effect of 12 fireworks types on the fold increase (over media control) in ROS activity in HPMEC cells treated with 100 μg/ml (PM10). The columns and error bars represent the mean and SEM, respectively. * p < 0.05 compared to media control. [file 12989_2020_360_MOESM1_ESM.pdf]
